# Transparent Biocompatible Polyelectrolyte Multilayer Coatings on Apples: Formation and Properties

**DOI:** 10.1021/acsfoodscitech.4c01027

**Published:** 2025-03-01

**Authors:** Katarina Jerin, Tin Klačić, Rajko Vidrih, Klemen Bohinc, Davor Kovačević

**Affiliations:** † Division of Physical Chemistry, Department of Chemistry, Faculty of Science, University of Zagreb, Horvatovac 102a, 10000 Zagreb, Croatia; ‡ Biotechnical Faculty, University of Ljubljana, Jamnikarjeva ulica 101, 1000 Ljubljana, Slovenia; § Faculty of Health Sciences, University of Ljubljana, Zdravstvena pot 5, 1000 Ljubljana, Slovenia

**Keywords:** polyelectrolyte multilayers, apples, chitosan, carboxymethyl cellulose, tensiometry, contact angle

## Abstract

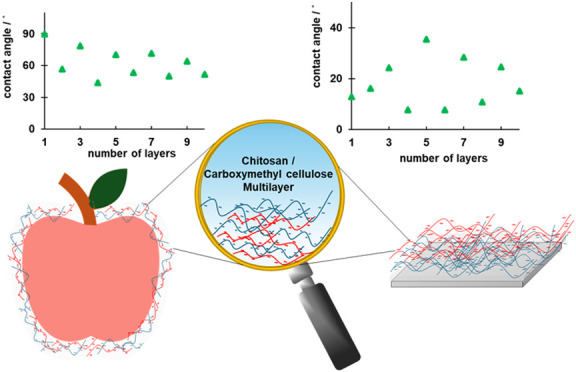

Polyelectrolyte multilayers (PEMs) are nanocoatings with possible applications in various areas, such as biomedicine and food technology. Recently, PEMs have been getting a lot of attention as potential food coatings for the prevention of fruit decay during transportation, storage, and shelf life. In this study, we fabricated thin films made of biocompatible polyelectrolytes, positively charged polysaccharide chitosan (CS), and negatively charged carboxymethyl cellulose (CMC) on apple surface and compared the results with the same multilayers formed on a model silica surface. The aim of our research is to correlate the fundamental aspects of the PEM build-up with their applications and to examine if contact angle measurements could be a useful tool for studying the formation of PEMs on apple surfaces. The influence of various experimental conditions on PEM formation was examined, and it was shown that the PEM build-up and properties such as thickness and hydrophobicity strongly depend on the applied experimental conditions (e.g., pH of the polyelectrolyte solutions). Moreover, for the first time we showed that the PEM build-up on apples could be verified using contact angle measurements. The most dominant zigzag pattern on both silica and apple surfaces at pH(CS) = 5.0 and pH(CMC) = 3.0 highlights the optimal conditions for multilayer formation and suggests that this process can be effectively monitored by using contact angle measurements. All of the results obtained in our study could serve as a basis for obtaining tuned biocompatible transparent polyelectrolyte multilayers on apples with optimized physicochemical properties, which could lead to the enhanced applications of the PEMs in the field of food technology.

## 1 Introduction

Polyelectrolyte multilayers (PEMs) are thin films formed by sequential deposition of oppositely charged polyelectrolytes onto a surface, allowing for the design of surface properties according to specific requirements. Potential applications of PEMs in industry, medicine, and biotechnology have been intensively investigated in recent years.^1−4^ One of the interesting possible applications of PEMs is food protection through thin coatings. Such coatings could play a crucial role in food safety, which in turn is strongly related to human health. For instance, alginate (AG) and chitosan (CS) PEM coating on fresh-cut melons effectively reduced microbial growth, including bacteria, yeasts, and molds, by creating a semipermeable antimicrobial barrier.^5^ Similarly, antioxidative and antibacterial self-healing edible PEM films composed of carboxymethyl cellulose (CMC) and CS have been demonstrated for fresh-cut apples, effectively inhibiting microbial growth and preventing oxidative browning, further enhancing food quality and safety.^6^ Moreover, flaxseed gum (FG) and CS based PEM coatings were shown to inhibit both internal microorganisms (molds and yeasts) and external pathogens such as *Escherichia coli* and *Staphylococcus aureus* on Mongolian cheese, extending food safety.^7^ These findings highlight the role of PEM coatings as versatile tools for protecting human health through microbial control. If we take into account that environmental pollution caused by nonbiodegradable plastic pollutants significantly influences various ecosystems, the development of novel functional and biodegradable films could be very useful. For example, it was shown recently^8^ that coatings based on corn starch and pectin containing zinc oxide nanoparticles exhibit processability, transparency, low water vapor permeation, and desirable mechanical properties for food packaging and coating applications.

In general, PEMs are formed on various surfaces primarily via electrostatic interactions between polycations and polyanions as the components of the film.^9,10^ However, hydrogen bonding, covalent bonding, or biospecific interactions could also play important role. The properties of PEMs (for example thickness, hydrophobicity or roughness) depend on various parameters such as the type of the substrate, the type of polycation/polyanion pair, and adsorption conditions.^11−15^ The most frequently adjusted coating conditions are pH, adsorption time, polyelectrolyte concentration, ionic strength, and type of salt added.^16,17^ Type of salt used to create PEMs can influence their hydration properties, a factor demonstrated to significantly impact cell adhesion. On the other hand, both the type and the concentration of salts can impact the chain dynamics and therefore self-healing, another desirable feature for food protection coatings. In addition to the above-mentioned experimental conditions, the presence of the initial layer, commonly referred to as the precursor layer, plays a significant role. Usually, poly(ethylenimine) (PEI) is used as the precursor layer for PEM build-up.^18−20^

When discussing biocompatible coatings, special emphasis must be given on the properties of the applied polyelectrolytes. Alongside other polyelectrolytes, chitosan (CS) emerges as a viable candidate for the fabrication of biocompatible PEMs. Chitosan is a typical biological macromolecule that recently attracted considerable interest as a natural antibacterial agent. Due to the protonation of chitosan’s amino groups in acidic solutions, it can effectively serve as a cationic polyelectrolyte. Up to date, in the process of PEM formation, chitosan has been combined with various polyanions,^21,22^ with special emphasis for different application in food industry^23,24^ and medicine.^25,26^ Since the preparation of suitable PEMs requires both a polycation and a polyanion, chitosan had to be paired with a biocompatible and nontoxic polyanion. In our study, we applied carboxymethyl cellulose (CMC), a derivative of naturally occurring cellulose. Among other desirable properties, CMC is relatively easily synthesized from cellulose, it is widely available, and has a broad range of applications as a biocompatible polymer.^27^ Studies on CS/CMC films have shown that this system displays self-healing properties that would be very desirable for the endurance of CS/CMC multilayers.^6,28^ In addition, we recently showed that in the case of CS/CMC films, using more concentrated CS solution over nonbiocompatible PEI as a precursor layer has shown a 2-fold increase in the film thickness.^29^

Additionally, it has to be stated here that chitosan is widely regarded as being a nontoxic, biologically compatible polymer.^30,31^ It is approved for dietary applications in various countries^32^ and by the FDA for use in wound dressings.^33^ On the other hand, it was also shown that CMC has no toxic effect at different doses on cellular structure,^34^ and these results support the use of CMC as food additive. Taking all of the findings mentioned above into account, CS/CMC multilayers could be a good choice for reliable, biocompatible coatings. For that purpose, edible coatings based on these polyelectrolyte multilayers should be applied, and the influence of added coatings on the properties of fruit surfaces should be assessed.

As substrate for studying biocompatible polyelectrolyte multilayer coatings, we chose apples. Apples are known for their favorable effects against various diseases, such as cardiovascular diseases, cancer, diabetes, and obesity. They are also a good source of quercetin, catechin, chlorogenic acid, and anthocyanin, all showing potential antioxidant and anti-inflammatory effects.^35^ When studying apples special emphasis should be given on the fruit cuticle which is the outermost layer that influences the apple’s response to environmental conditions.^36^ In addition to apple peel’s chemical composition, its surface properties might be important quality parameters. Properties such as roughness, hydrophobicity, and surface charge play important roles in microbial colonization. Especially hydrophobicity should be taken into account since the contact angle depends on the presence of nonpolar wax. In the case of higher wax content, higher contact angle values are obtained. That property is related with a significant challenge which is the confirmation of the formation of PEMs on apple surfaces, and contact angle measurements could be a useful alternative. The reason therefore is that conventional methods used for determination of PEM formation and thickness such as ellipsometry, optical reflectometry, or QCM are not suitable for fruit surfaces due to high roughness, low reflectivity, and instability of such surfaces.

The aim of this manuscript is, initially, to examine the impact of the pH value of polyelectrolyte solutions (i.e., chitosan and CMC solution) on the formation and properties of polyelectrolyte multilayers built-up on typical metal oxide surface. Furthermore, the present investigation aims to go a step further by investigating the formation and properties of polyelectrolyte multilayer coatings on fruit surface (in this case apples). One of the challenges in monitoring the formation of PEMs on surfaces such as apples is to find the appropriate method that can be applied on such surfaces. Therefore, last but not least, the objective is to apply contact angle measurements as a new tool for studying polyelectrolyte multilayer growth on food surfaces such as apples.

## 2 Experimental Section

### 2.1 Substrates

Polished silicon wafers were cut from larger plates (Nano-Tec, ⟨100⟩ orientation, P-doped with boron) to 1 cm × 1 cm for ellipsometric and atomic force microscopy (AFM) measurements, while for the contact angle measurements, the plates were cut to 1 cm × 3 cm size. The plates were cleaned in a “piranha” solution for 1 h. The piranha solution was prepared from concentrated H_2_SO_4_ (Gram-mol) and 30% H_2_O_2_ solution (Merck) in volume ration 3:1, continuously stirred, and heated to approximately 70 °C. The plates were rinsed with deionized water (Milipore Milli-Q Plus 185 purification system, Merck KGaA, Germany, conductivity 0.055 μS cm^–1^) and dried before adsorption with a stream of argon gas (Argon 5.0, Messer). It should be noted here that a thin oxide layer is spontaneously formed on silicon surface by oxidation of Si with oxygen from air and is reported to be around 2 nm thick.^19^

The apples (cultivar “Idared,” producer Eurosad Krško, Slovenia) were in optimal edible maturity stage; their physicochemical properties were as follows: firmness (5.1 ± 0.5) kg, soluble solids (13.5 ± 0.03) % Brix, titratable acidity (3.11 ± 0.03) g dm^–3^, and pH value 3.83. CIE color parameters measured on green and red parts of fruit amounted to as follows: L* 74.4 ± 2.8, a* – 11.1 ± 1.3, and b* 44.7 ± 1.0 for the green part of apple and L* 38.2 ± 2.9, a* 35,8 ± 1.1 and b* 19,8 ± 1.9 for the red part of apple. For contact angle measurements, the apples were thoroughly cleaned in water, rinsed with deionized water prior to drying, and then cut to the size of sample holder (approximatively 1 inch in diameter), leaving the surface of the apple peel intact. The rinsing step was repeated to ensure the complete removal of any juice residue from its surface.

### 2.2 Polyelectrolyte Electrophoretic Mobility

The electrophoretic mobility of polyelectrolytes in solution was measured using the electrophoretic light scattering technique with a Zetasizer Pro (Malvern Panalytical, United Kingdom), and the data were obtained with ZX Xplorer software (ver. 3.0.0, Malvern Panalytical, United Kingdom). To measure electrophoretic mobility, an alternating electric field was applied between electrodes with a maximum voltage of ±30 V. All measurements were performed in a disposable folded capillary cell (DTS1070, Zetasizer Nano series). Measurements were conducted at the same pH as the polyelectrolyte solutions used for the preparation of PEM. Each sample was measured 3 times, with 10 to 15 runs per measurement and a 5 s pause between subruns. The error associated with each data point was calculated based on the standard deviation of three measurements. The water parameters, index of refraction (*n* = 1.33) and viscosity (η = 0.8872 mPa s) at 25 °C, were taken into account for the dispersant medium.

### 2.3 Polyelectrolyte Multilayer Formation

Polyelectrolytes used for the adsorption were chitosan (low-molecular weight 50–190 kDa) and carboxymethyl cellulose sodium salt (medium viscosity), both obtained from Sigma-Aldrich and used as received from the supplier. Polyelectrolyte multilayers were prepared using the conventional layer-by-layer (LbL) method,^9^ where each adsorption step lasted 5 min, followed by three rinsing steps of 2, 1, and 1 min again. Before each measurement, PEM was dried in a stream of argon. The polyelectrolyte solutions were prepared by dissolving the appropriate amount in either 1% acetic acid (Kefo) (CS) or water (CMC) and left to dissolve for 1 day. The first layer was deposited from the CS solution with concentration of 15 g dm^–3^ while every other step of adsorption was performed from polyelectrolyte solution of concentration 1 g dm^–3^. This process was repeated until the planned number of layers (ten) was deposited onto the substrate. To investigate the dependence of the thickness of PEM on the pH of polyelectrolyte solutions, the pH was adjusted by adding either a solution of HCl (Merck) (*c* = 1.0 mol dm^–3^) or NaOH (Merck) (*c* = 1.0 mol dm^–3^). The pH of 15 g dm^–3^ CS solution was not adjusted due to its high viscosity. The pH meter (913 pH Meter, Metrohm, Switzerland) equipped with a combined glass microelectrode (Metrohm, Switzerland) was calibrated with 4 standard buffers (pH 4.01, 7.00, 9.21, and 10.01, *Hamilton*)

### 2.4 Ellipsometry Measurements

Thickness of polyelectrolyte multilayers after deposition of each polyelectrolyte layer was measured on an L116B-USB ellipsometer (Gaertner Scientific Corporation, USA). The ellipsometer is equipped with single wavelength He–Ne laser of 632.8 nm with incident angle of 70°. All measurements were performed under ambient conditions (25 °C). Data processing was performed by the Gaertner Ellipsometric Measurement Program (Version 8.071, Gaertner Scientific Corporation). To determine the PEM thickness, a tree-box model was used. In this model, the air is considered an optical continuum (*n* = 1.00), PEM-multilayer (*n* = 1.46) as one-phase system and Si/SiO_2_ as a substrate. To determine the refractive index of the substrate, measurements were conducted at ten different locations on the substrate. Similarly, the thickness of the PEM was determined by conducting measurements at ten different locations. The error bars indicate the standard error for each data point.

### 2.5 Atomic Force Microscopy Measurements

The influence of the pH of the polyelectrolyte solution on the surface properties of polyelectrolyte multilayers, including film thickness, root mean-square roughness (*R*_q_), and topography, was investigated using atomic force microscopy (AFM). Measurements were conducted with a Bruker Multimode 8E AFM apparatus (Bruker, USA) in tapping mode, employing NCHV probes. The cantilever probe had a nominal resonant frequency of 320 Hz and a spring constant of 40 N/m. The rectangular tip of the cantilever featured a nominal curvature radius of 8 nm and measured 10–15 μm in height. All measurements were analyzed in Nanoscope Analysis 2.0 software (Version 2.00, Bruker). During the measurements, the 5 μm × 5 μm area was scanned at the rate of 1 Hz to create a picture resolution 512 pixels × 512 pixels. To remove the tilt and bow, first and second order flattening was employed. In order to perform the adequate analysis, the surface roughness was determined at five different locations on the surface of each sample. Standard error reported here were determined for five measurements. The thickness of PEM film was determined by scratching the surface with sharp tweezers. Creating a scratch removes part of PEM, and the sharp line enables the analysis of the height difference between the substrate and the PEM’s surface. The *R*_q_ and topography of the apple skin as well as of the PEM-coated apple skin were determined using the same setup. Both apple skin and apple skin coated with PEMs were carefully cut from the apple sample and glued to the piece of paper and then directly attached to the sample holder. For the determination of the *R*_q_, three AFM images of the surface, each measuring 25 μm × 25 μm, were captured. Additionally, AFM images of 5 μm × 5 μm were acquired to provide a more detailed representation of the studied surfaces. In total, three different samples were examined: apple skin without PEM, apple skin with PEM built-up under conditions of pH(CMC) = 3.0 and pH(CS) = 5.0, and apple skin with PEM built-up under conditions of pH(CMC) = 5.0 and pH(CS) = 3.0.

### 2.6 Contact Angle Measurements

**Figure 1:**
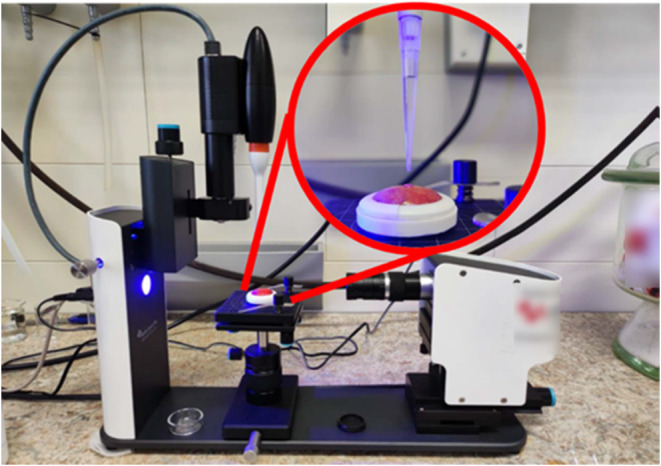
Tensiometer used for water contact angle measurements on the apple’s surface. An apple peel sample is placed on a custom support designed to ensure an unobstructed view during the measurement of the water contact angle.

Water contact angles for both apple skins and silicon wafers were determined by using the sessile drop method with a Theta light optical tensiometer (Bioline Scientific, Finland) equipped with a USB3 camera. The resolution was set to 1280 × 1024 with 203 frames per second. All measurements were conducted with deionized water under ambient conditions (25 °C). The volume of the droplet was set to 5 μL. The droplet profile on the surface was recorded for 10 s. To minimize contact angle hysteresis resulting from both the initial drop and evaporation, frames from the third to the sixth second were used. The water contact angle reported here is the mean value of the left and right contact angle and represents the mean of 62 measurements. The data were analyzed using One Attension software with the Young–Laplace model. For each sample and polyelectrolyte layer, five different contact measurements were taken. For each data point, the standard error has been calculated. To perform the contact angle measurements, a piece of apple was carefully cut and placed on a custom-made support designed specifically for this purpose. Great care has been taken both to prevent damage to the apple skin and to ensure an unobstructed view for the camera. The support had to accommodate the apple’s natural curvature without being overly convex, which could have affected the accuracy of the measurements. On the other hand, if the apple were too flat, the skin would rapture. The support was crafted from Teflon and included a poly(vinyl chloride) (PVC) pipe rosette resembling a clip-in button. This design not only ensured the correct curvature for the apple’s surface but also maintained a clear camera view. The support was also designed to be compatible with the LbL process, allowing for multiple dipping and rinsing steps. An example of the tested apple sample is shown in Figure 1.

### 2.7 Spectrophotometric Measurements

The vis spectrum of uncoated and PEM-coated apples was obtained with a Cary 5000 ultraviolet–visible (UV–vis)-NIR spectrophotometer equipped with an internal diffuse reflectance accessory (Agilent Technologies, USA). Spectra were acquired at ambient conditions from 400 to 800 nm in reflectance mode. Total reflectance was sampled at 1 nm intervals with an integration time of 0.1 s. Before the spectrum of the samples was collected, zero/baseline correction was performed with a poly(tetrafluoroethylene) reference disk. The obtained reflectance spectra were processed in Cary WinUV software (version 6.4.0.1610, Agilent Technologies).

## 3 Results and Discussion

As mentioned in the introduction, this study consists of two parts. In the first part, we investigated the properties of the examined polyelectrolytes as well as the preparation and properties of CS/CMC multilayers formed on the silicon substrate. In the second part, on the other hand, we investigated the formation of the aforementioned CS/CMC multilayers on apples and their properties.

### 3.1 Electrophoretic Mobility of CS and CMC

Contrary to strong polyelectrolytes, the charge of weak polyelectrolytes is significantly influenced by the pH of their solutions. The charge of polyelectrolytes plays an important role in multilayer formation, affecting properties of the film such as film thickness, surface roughness, wettability, and growth regime.^37−39^ Chitosan and carboxymethyl cellulose are weak polyelectrolytes, and their degree of dissociation is influenced by changes in pH. In this study, we used electrophoretic mobility measurements to evaluate the degree of the polyelectrolyte charge at different pH (Table 1). The degree of dissociation is related to the protonation or deprotonation of their functional groups along the polyelectrolyte chain. Depending on the concentration of H^+^ or OH^–^ ions in the solution, the −COOH group of CMC (p*K* ≈ 4.0)^40^ and the −NH_2_ group of CS (p*K* ≈ 6.5)^40^ will be dissociated. As shown in Table 1, the electrophoretic mobility of CS decreases as pH increases. On the other hand, as expected, the electrophoretic mobility of CMC decreases as pH decreases.

**Table 1:** Electrophoretic Mobility of Polyelectrolytes CS and CMC at Different pH Values

	pH = 3.0	pH = 5.0	pH = 6.0	pH = 7.0
	μ/μm cm V^–1^ s^–1^	μ/μm cm V^–1^ s^–1^	μ/μm cm V^–1^ s^–1^	μ/μm cm V^–1^ s^–1^
CS	3.244 ± 0.287	1.593 ± 0.075	1.284 ± 0.092	not measured
CMC	–1.19 ± 0.083	–2.547 ± 0.574	not measured	–3.842 ± 0.172

### 3.2 Characterization of CS/CMC Nanofilms on Silica Surface

#### 3.2.1 Growth and Thickness of CS/CMC Nanofilms

**Figure 2:**
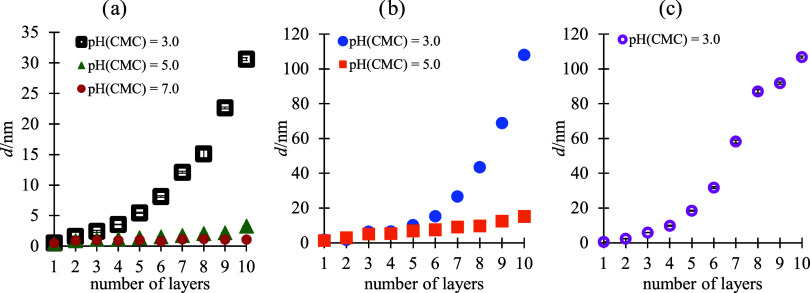
Ellipsometric thickness of CS/CMC multilayers built-up at pH (CS) (a) 3.0, (b) 5.0, and (c) 6.0 and different pH values of CMC solution as a function of the numbers of deposited polyelectrolyte layers on the Si substrate. The data points are visually larger than the error bars due to the scaling.

In order to determine the optimal conditions for multilayer build-up, we systematically examined the formation of chitosan/carboxymethyl cellulose multilayers. The formation process was followed by ellipsometric measurements while the total thickness of (CS-CMC)_5_ multilayer was determined additionally by AFM. We started with keeping the pH value of chitosan solution at pH = 3.0 and varying the pH of carboxymethyl cellulose solution (Figure 2a). At pH = 3.0, nearly all of the −NH_2_ groups of chitosan should be protonated, resulting in the polymer carrying a high positive charge, as confirmed by electrophoretic mobility measurements. Changing the pH of carboxymethyl cellulose from pH = 3.0 to pH = 5.0 and then to pH = 7.0 allows us to evaluate the influence of the polymer’s charge on the growth regime and thickness of the multilayer. Later on, we examined the formation of the multilayer in the case of chitosan solution at pH = 5.0 (Figure 2b) and pH = 6.0 (Figure 2c), where the polymer’s (CS) groups are less protonated.

Multiple conclusions can be drawn from the results presented in Figure 2. First of all, it can be seen that the CS/CMC multilayer grows exponentially only when pH(CMC) = 3.0. At this pH, electrophoretic mobility measurements have shown that carboxymethyl cellulose is very weakly charged. In all other cases, the growth of the polyelectrolyte multilayer is predominantly linear. From this result, it could be concluded that in the case of CS/CMC multilayers, predominant electrostatic interactions lead to thin adsorbed layers. A high degree of dissociation makes the polymer chain stiff, hindering the formation of loops and hoops on the multilayer surface. Furthermore, the linear growth of the multilayer suggests an intrinsic compensation between polyelectrolytes in adjacent layers. The intrinsic pairs formed between the polyelectrolytes restrict the movement of the polyelectrolyte chain and multilayer build-up. It is important to point out that the thickness of film with 10 layers at pH(CS) = pH(CMC) = 3.0 is significantly less pronounced (*d* = 30.6 nm) than at higher pH values of chitosan solutions (*d*(pH = 5.0) = 108.0 nm and *d*(pH = 6.0) = 106.8 nm) with pH(CMC) = 3.0. Under applied chitosan solution conditions, electrophoretic mobility measurements indicate a low degree of protonation, which shows that the forces driving the multilayer build-up are not purely electrostatic. The exponential growth (Figure 2) can be explained by the formation of hydrogen bonds between the −NH_2_ group of CS and the −COOH group of CMC, which allows the polyelectrolytes to diffuse more than in the case of electrostatic pairing. This is in agreement with our recent paper^29^ but also with the study performed by Tirrell and co-workers^37^ who found out that poly(allylamine hydrochloride) and poly(acrylic acid) polyelectrolyte complexes can establish hydrogen bonding at low pH values. For several other systems, hydrogen bonding has also been reported as crucial for the polyelectrolyte multilayer build-up.^38,39^

**Figure 3:**
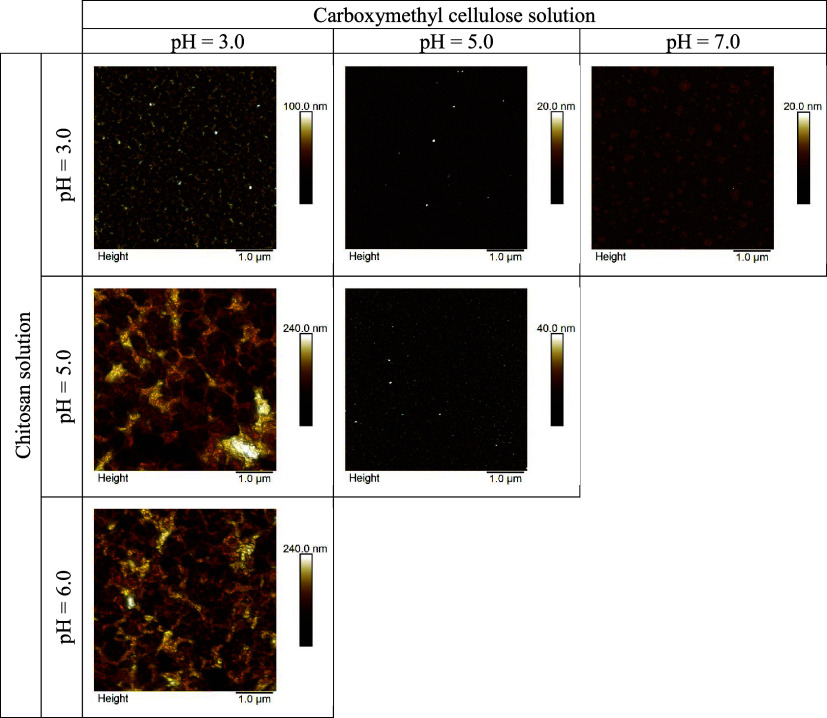
AFM images (5 μm × 5 μm) of Si/SiO_2_ substrates with built-up (CS/CMC)_5_ multilayers formed from polyelectrolyte solutions having various pH values.

As a rule, the thickness of the polyelectrolyte multilayer determined by ellipsometry is similar to the thickness determined by AFM. Only in the case when pH(CS) = 6.0 and pH(CMC) = 3.0, the film thicknesses are not comparable (Table 2). At the same time, under these conditions, the surface *R*_q_ roughness of (CS/CMC)_5_ is the highest (Figure 3). Surface roughness can affect the result of ellipsometric measurements due to diffuse light scattering, potentially reducing the precision of the measurements. On the other hand, AFM measurements determine the thickness on a relatively small surface, while ellipsometric measurements can determine the thickness on a larger surface of the sample.

**Table 2:** Thickness of the Polyelectrolyte Multilayers (CS/CMC)_5_ on Si/SiO_2_ Determined by AFM and Ellipsometry

pH(CS)	pH(CMC)	*d*(AFM)/nm	*d*(ellipsometry)/nm
3.0	3.0	32.6	30.6
3.0	5.0	3.1	3.3
3.0	7.0	1.0	1.1
5.0	3.0	118.0	108.0
5.0	5.0	13.8	15.2
6.0	3.0	165.0	106.8

#### 3.2.2 Surface Roughness and Morphology of CS/CMC Nanofilms

Atomic force microscopy was also used in this part of the research as a complementary method to ellipsometry for determining the thickness of the multilayer as well as for studying the morphology of the film surface characterized by the surface roughness. Therefore, we analyzed all obtained AFM images using Nanoscope Analysis 2.0 to determine Root mean square roughness, *R*_q_, and thickness of examined nanofilms. All (CS/CMC)_5_ films prepared for ellipsometric measurements were characterized also by atomic force microscopy.

The morphology of the multilayers significantly differs depending on the pH of the polyelectrolyte solutions. In conditions where thin films form, the surface of the silica substrate is covered in worm-like structures resembling the skin of a cantaloupe melon (Figure 3, pH(CS) = 3.0 and pH(CS) = pH(CMC) = 5.0). As the film thickness increases, these surface features become more pronounced with the most pronounced cantaloupe resemblance for the case of pH(CS) = pH(CMC) = 3.0. Only in the cases pH(CS) = 3.0 and pH(CMC) = 7.0 is a granular structure observed. In contrast, thick films (thickness higher than 100 nm) show irregular morphology with less ordered structures (pH(CS) = 6.0 and pH(CS) = 5.0 when pH(CMC) = 3.0). The trend is observed when both carboxymethyl cellulose and chitosan are deprotonated and have low electrophoretic mobility (Table 1).

**Figure 4:**
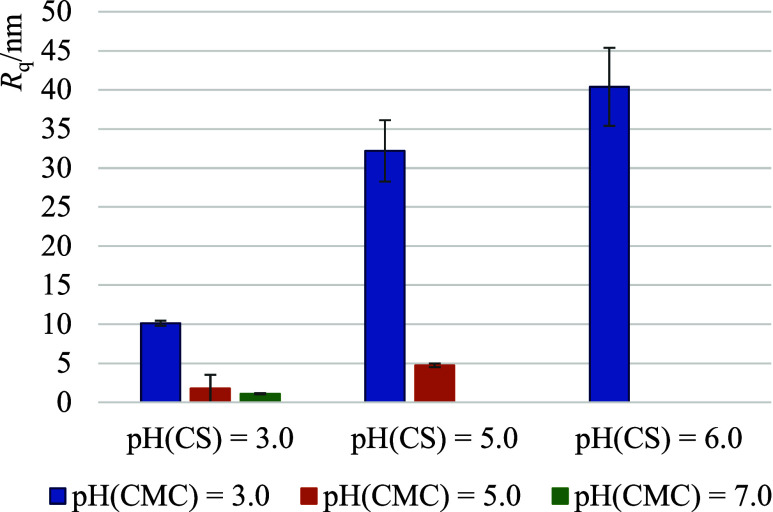
Root mean square roughness, *R*_q_, of the (CS/CMC)_5_ polyelectrolyte multilayer built-up on Si/SiO_2_ substrate at various pH values of polyelectrolyte solutions.

It was also shown that the surface roughness, *R*_q_, (Figure 4) is higher for systems in which pH(CMC) = 3.0, which is consistent with the thickness of the multilayer (Table 2), and the roughness, *R*_q_, increases with the increase in the pH of the chitosan solution. This phenomenon can be explained by lower association among CS and CMC at low electric charge densities of both polymers and fewer adhesion point resulting in loopy conformation.^40^ Therefore, it is evident that the pH of the polyelectrolyte solution significantly influences the surface roughness and surface morphology of the CS/CMC polyelectrolyte multilayer.

#### 3.2.3 Contact Angle of CS/CMC Nanofilms

**Figure 5:**
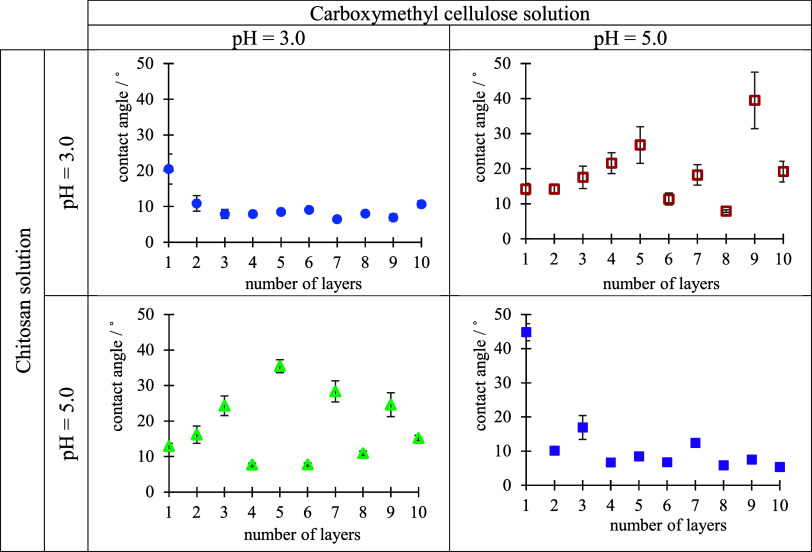
Water contact angles determined during build-up of CS/CMC multilayer on the Si/SiO_2_ substrate presented as a function of the number of layers. PEMs were prepared at various pH values of polyelectrolyte solutions. Odd numbers represent positively charged films with CS as the outermost layer, whereas even a number of films have negatively charged CMC as the outermost layer. The data points are visually larger than the error bars due to the scaling.

The contact angle of a water droplet on various surfaces can be measured by using optical tensiometry. The contact angle is a measure of the hydrophobicity of the surface; therefore, a larger contact angle indicates a more hydrophobic surface. It is known that if the water contact angle is larger than 90°, surfaces are hydrophobic, while surfaces with contact angles lower than 90° are hydrophilic. Numerous factors influence the change in the wettability of the surface. Chemical composition of the adsorbed polyelectrolyte and hydrophobicity of its functional groups are two examples of such factors in the case of polyelectrolyte adsorption on various surfaces. As a rule, surfaces covered with polycations are more hydrophobic than those covered with polyanions.^39,41^ The successive adsorption of polycations and polyanions should lead to the continuous change in the measured contact angle due to the change in hydrophilicity of their functional groups.^42,43^ Consequently, the obtained zigzag pattern, reflecting changes in surface hydrophobicity after each adsorption cycle, can be considered as evidence of polyelectrolyte adsorption and PEM formation. Figure 5 presents change of water contact angle during CS/CMC multilayer formation on the silica surface from polyelectrolyte solutions of various pH values. In general, in the case of CS being the outermost layer, the water contact angle exhibits a higher value, indicating more hydrophobic character of the multilayer. In contrast, in the case of CMC, as polyanion, being the outermost layer, the multilayer tends to be more hydrophilic resulting in lower contact angles. In our case, such a pattern is the most dominant when on the silica surface polyelectrolyte multilayers were built-up under the conditions pH(CS) = 5.0 and pH(CMC) = 3.0. Such changes in the hydrophobicity, or the lack thereof in other cases, can be explained by the fact that under these conditions, the polyelectrolyte multilayer is the thickest and grows the fastest, which leads to greater surface coverage. Greater surface coverage with PEMs reduces the influence of the substrate on the contact angle.^44^

### 3.3 Characterization of CS/CMC Nanofilms on Apple Surface

#### 3.3.1 Contact Angle Measurements

**Figure 6:**
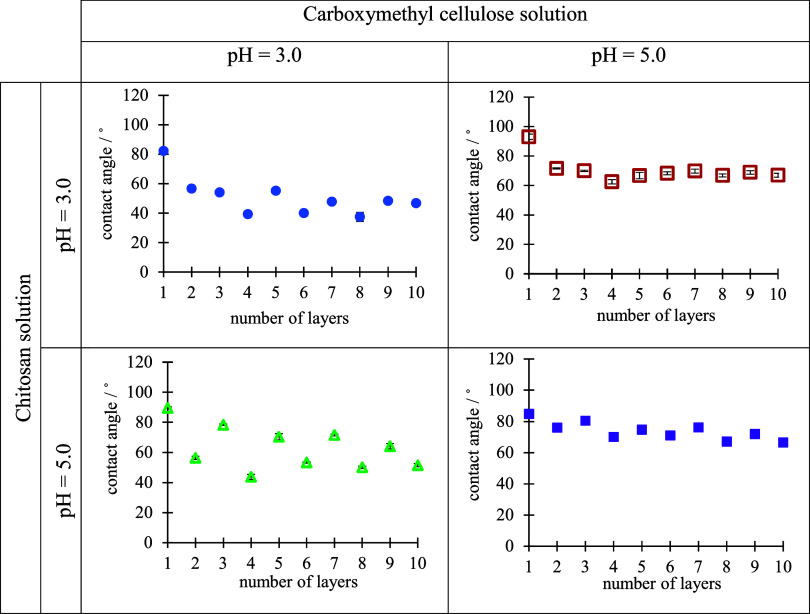
Water contact angles determined during build-up of CS/CMC multilayers on “Idared” apple surface presented as a function of the number of layers. PEMs were prepared at various pH values of polyelectrolyte solutions. Odd numbers represent positively charged films with CS as the outermost layer, whereas even number films have negatively charged CMC as the outermost layer. The data points are visually larger than the error bars due to the scaling.

As stated in the introduction, one of the aims of our study was to apply contact angle measurements as a new tool for studying polyelectrolyte multilayer growth on food (e.g., apple) surfaces. The contact angle results obtained earlier for uncoated autochthonous apple cuticles^45^ showed that such surfaces were, in general, hydrophilic, with contact angles lower than 85°. On the other hand, commercial apple cuticles were found to be more hydrophobic, with contact angles greater than 90°. In our case, the contact angle of the uncoated apple was found to be around 100°. The reason for such a behavior is that commercial apple surfaces contain several wax groups that increase the contact angle. The results obtained for the determination of the contact angle during the process of polyelectrolyte multilayer formation on the examined “Idared” apple surface at different pH conditions are presented in Figure 6. The zigzag pattern, after each adsorption step, is the most dominant on the surface of the apple under the conditions of pH(CS) = 5.0 and pH(CMC) = 3.0, as previously observed on the silica surface. The dominant zigzag pattern in the case of the silica surface was explained by the increased thickness accompanied by more complete surface coverage. Although thickness values of the PEMs were not determined on the apple surface, the pronounced zigzag pattern under these pH conditions suggests that a similar process of surface coverage is occurring. This can be considered evidence of effective multilayer formation on the apple surface.

#### 3.3.2 Surface Roughness and Morphology of CS/CMC Nanofilms on Apple Skin

AFM measurements were conducted on apple surfaces to complement the previously reported data obtained on the silica substrates. In that manner, three samples were examined: (1) an apple surface without PEM, (2) an apple surface coated with a PEM prepared from PE solutions with pH(CS) = 5.0 and pH(CMC) = 3.0, and (3) an apple surface coated with a PEM prepared from PE solutions with pH(CS) = 3.0 and pH(CMC) = 5.0. These samples were selected due to the significant differences in PEM thickness (Figure 2 and Table 2).

**Figure 7:**
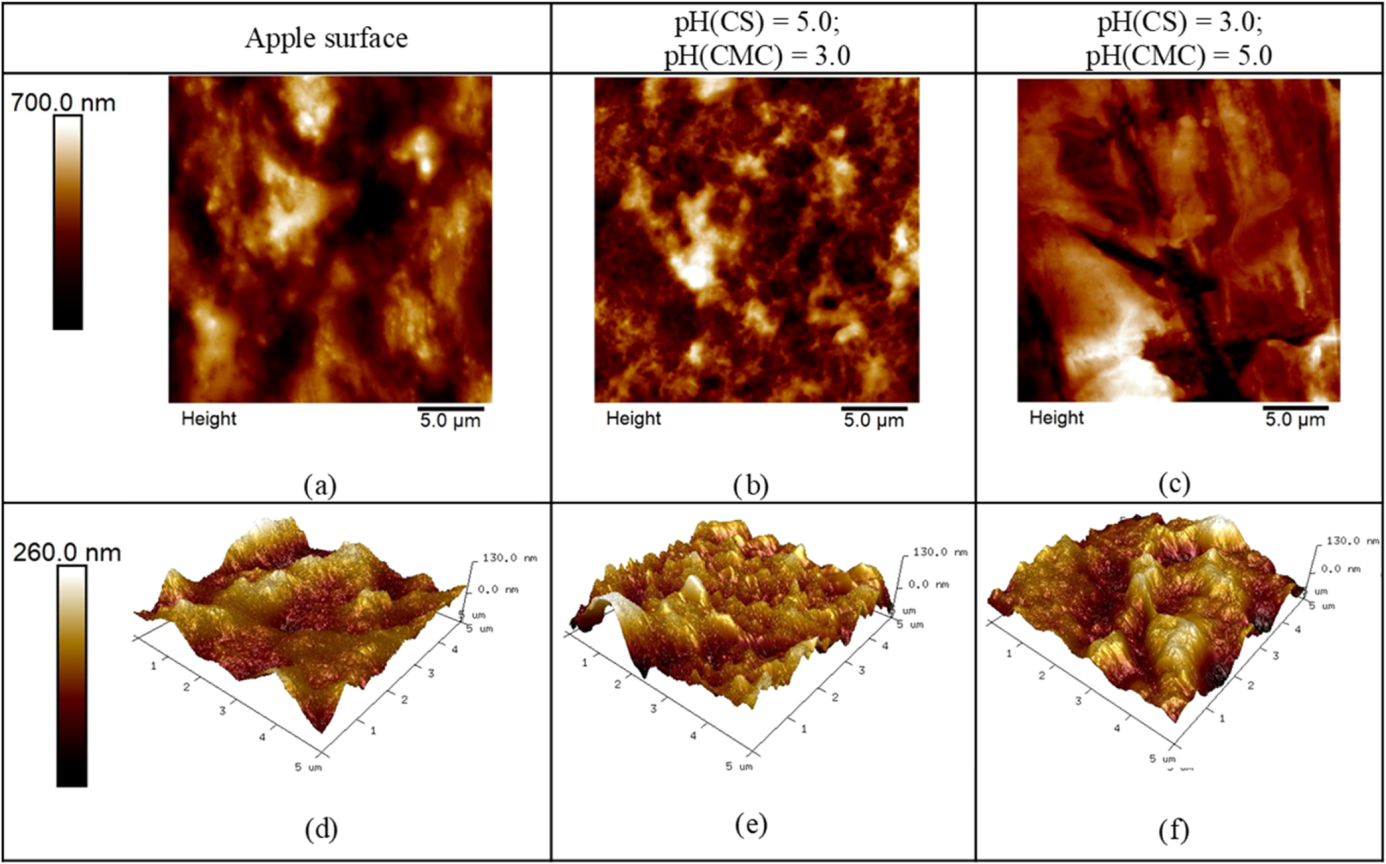
25 μm × 25 μm AFM images of (a) apple surface (b, c) apple surface with built-up (CS/CMC)_5_ multilayers formed from polyelectrolyte solutions having various pH values and (d–f) corresponding 5 μm × 5 μm 3D AFM images.

The presence of the PEM coating prepared on the apple surface from PE solutions with pH(CS) = 5.0 and pH(CMC) = 3.0 can be clearly detected by comparing the surface morphology of the bare apple skin (Figure 7a) and apple skin coated with such a film (Figure 7b). The observed morphology of PEM closely resembles the one observed under the same pH conditions on the silica surface (Figure 3). In contrast, the much thinner PEM prepared from PE solutions with pH(CS) = 3.0 and pH(CMC) = 5.0 can hardly be distinguished from apple skin (Figure 7c), even at a smaller scale (Figure 7e).

Similar surface roughness (*R*_q_) values were obtained for the bare apple skin (*R*_q_ = 149 nm ± 17 nm) and the surface with thinner PEM (*R*_q_ = 172 nm ± 19 nm), while a significantly smoother surface was observed for the thicker PEM (*R*_q_ = 90 nm ± 4 nm). Interestingly, this trend is opposite to what was previously observed for the same PEMs on silica surfaces. This difference should not be attributed to the PEM morphology itself but rather to the inherently distinct roughness of the silica and apple surfaces. It is evident that a thin PEM cannot fill the surface irregularities of the rough apple skin as effectively as a thicker film can. This conclusion is consistent with the literature,^46^ and it indicates that PEM has the ability to smooth initially rough surface.

#### 3.3.3 Transmittance of PEM Coating on Apples

**Figure 8:**
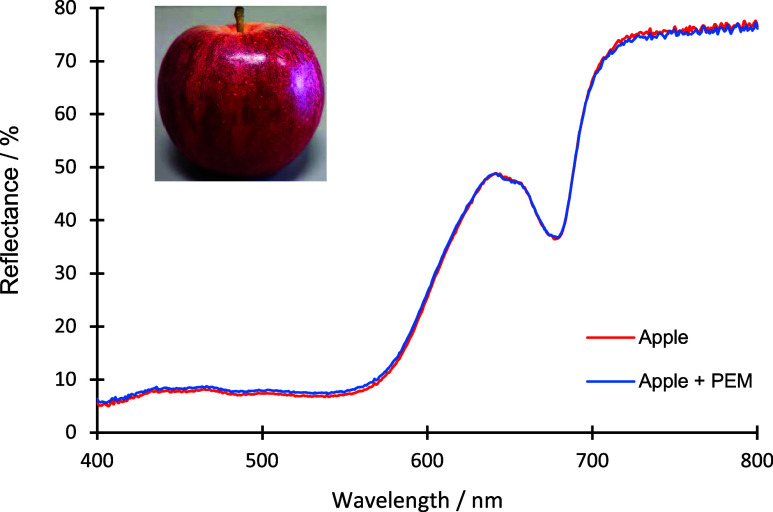
Reflectance spectrum of uncoated apple (red line) and apple coated with a (CS/CMC)_5_ multilayer at pH(CS) = 5.0 and pH(CMC) = 3.0 (blue line). Inset shows optical photography of the apple used in experiments.

The optical properties of PEM coatings are also very important for food protection applications with the transparency of thin films as a very desirable property. If the coating on the apples were, for example, brown, it could cause repulsion among customers and financial losses. One way to investigate the optical properties of solid samples is with a spectrophotometer equipped with a diffuse reflectance accessory. An instrument of that kind is typically used for studying the optical properties of antireflective and antifogging coating;^47^ however, it can also be used for food samples. Figure 8 shows the reflectance spectrum of the uncoated apple and apple coated with the (CS/CMC)_5_ multilayer at optimal conditions (pH(CS) = 5.0 and pH(CMC) = 3.0), as established by contact angle measurements.

Three spectral regions characterize the reflectance spectrum of the uncoated apple. In the 400–575 nm region, the reflectance of the apple surface is low (around 7%). Then, in the 575 to 675 nm region, an increase of reflectance can be observed with the maximum of reflectance at 645 nm. In the last region (from 675 to 800 nm), the reflectance is high, between 70 and 80%. The color of solids strongly depends on reflected visible light from their surfaces. As the reflectance of the apple is highest in the 675 to 800 nm region, the corresponding color of the apple is red (inset in Figure 8). Figure 8 also shows the reflectance spectrum of apples coated with PEM. The spectrum of the PEM-coated apple overlaps with the spectrum of an uncoated apple, which means that PEM is transparent to visible light. Therefore, we conclude that modifying the apple surface with the (CS/CMC)_5_ multilayer did not affect the food appearance.

The main idea of this study was to find a way to determine the formation of biocompatible polyelectrolyte multilayers on apple surfaces and to compare the properties of the obtained PEMs with properties of the same PEMs built on silica substrates. In the first stage of this study, we focused on the effect of pH on the fabrication of the PEMs on silica. Systematic experimental investigation enabled us to find out the properties (thickness, roughness, and hydrophobicity) of all studied PEMs. In the second part of this study, we prepared and characterized PEMs on the apple surfaces. Special emphasis was given to the determination of the contact angle of all layers in the process of the formation of PEMs on apple surfaces. It is worth mentioning here that there are no results in the literature dealing with characterizing the growth of PEMs on apples by means of contact angle changes. The presented results confirm that contact angle measurements can be used to monitor the formation of polyelectrolyte multilayers on the surface of apples, which has not been reported in the literature for similar samples. Also, by variation of the experimental conditions, such as the pH of the polyelectrolyte solution, it is possible to prepare polyelectrolyte multilayers with different properties, which will also affect the properties, of apples coated with polyelectrolytes. To sum up, our results provide a framework for obtaining tunable, completely biocompatible chitosan-carboxymethyl cellulose nanofilm coatings on apples with optimized physicochemical properties for applications in food technology. Moreover, such an approach could be extended to other types of biocompatible food coatings and various types of food surfaces. That could be, for example, additional types of fruit surfaces (various apple cultivars but also other fruit) as well as other polyelectrolytes that can be applied as coatings. Moreover, the experimental conditions for the formation of polyelectrolyte multilayers could be varied, which could lead to the formation of coatings with different properties.
